# Hyaluronic Acid Filler Injection Guided by Doppler Ultrasound

**DOI:** 10.1055/s-0043-1770078

**Published:** 2023-08-02

**Authors:** Won Lee

**Affiliations:** 1 Yonsei E1 Plastic Surgery Clinic, Anyang, South Korea

**Keywords:** soft tissue filler, hyaluronic acid filler, ultrasound, filler complication

## Abstract

Doppler ultrasound can be used to detect almost all arteries of the face before injecting the hyaluronic acid (HA) filler. The relatively more dangerous sites of filler injection are the glabellar wrinkle, forehead, temple, nose, and nasolabial fold area, and it is recommended to map the vasculature of these areas by Doppler ultrasound before performing filler injection. The Doppler ultrasound detection method is included as a video. Internal carotid arterial branches, the supratrochlear, supraorbital, and dorsal nasal arteries, and external carotid arterial branches, the superficial temporal and facial arteries, are very important arteries when injecting HA filler; thus, Doppler ultrasound detection is recommended.

## Introduction

The use of soft tissue fillers is one of the most commonly used aesthetic procedures for facial rejuvenation. 1 Although filler injections are easy to perform, they have complications. 2 Vascular complications, such as skin necrosis and ocular complications, are the most serious complications of filler injections. 3 To avoid these, knowledge of facial anatomy is key. However, the vasculature varies between individuals, and injectors cannot locate all the vasculature during filler injection. Doppler ultrasound can detect vessels of the face by detecting the interaction of sound waves with tissue. Ultrasonography is a well-known and safe procedure that can be applied to minimally invasive procedures of the face. 4 Ultrasound examination can be used to map vasculature and detect the locations of previous fillers or fillers when vascular complications occur. 5 Recently, real-time ultrasound-guided filler techniques have been developed. 6 However, it is very difficult to perform real-time detection and filler injection concomitantly because of the number of hands needed, delicate filler injection procedures, and the possibility of infection. In this review article, we summarize previous studies on Doppler ultrasound detection of arteries of the face and hyaluronic acid (HA) filler injection to promote the implementation of safer procedures. All patients provided written informed consent for publication of photograph.

## Ultrasound Wave Frequency

**Fig. 1:**
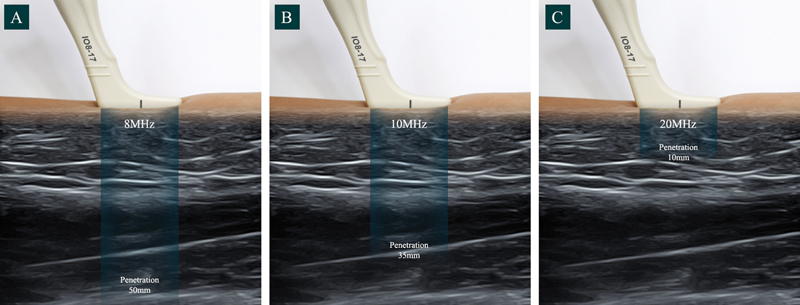
Different depths of visualization with different frequencies: ( **A** ) 8 MHz, ( **B** )10 MHz, and ( **C** ) 20 MHz.

In our study, an 8 to 17 MHz hockey stick probe (E-cube, Alpinion Medical Systems Co., South Korea) was used. The use of 8 MHz provided a depth of visualization of approximately 50 mm, while 20 MHz, gives a depth of 10 mm ( Fig. 1 ). Thus, 8 to 17 MHz probe can cover all the facial layer detection. Doppler ultrasound can detect both arteries and veins and distinguishes between them by the way blood circulation changes when the probe presses on the vessel. If blood circulation completely stops, the vessel is identified as a vein. If blood flow persists, it indicates an artery. Thus, artery and vein cannot be distinguished by color but distinguished by probe pressing.

## Glabellar Wrinkle Correction

**Fig. 2:**
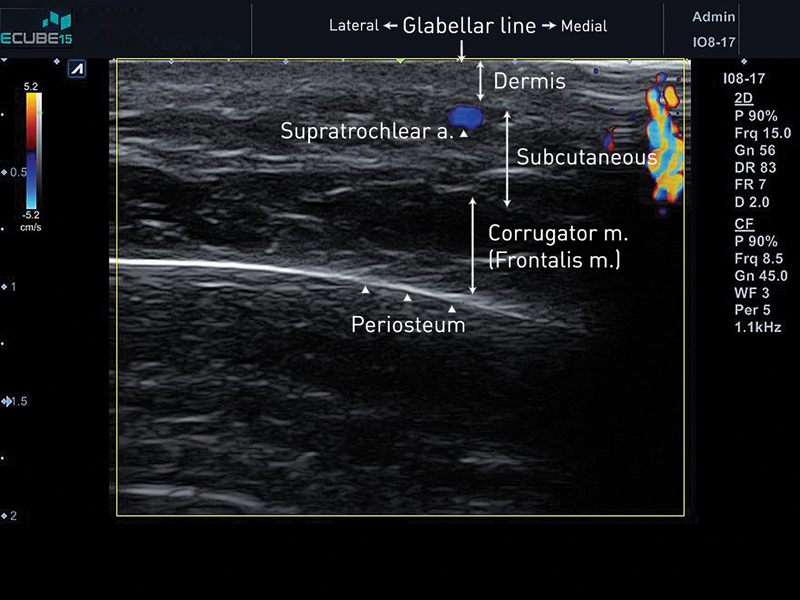
The supratrochlear artery is located just beneath glabellar wrinkle dermal layer.

One of the dangerous areas for filler injection is the glabellar area where the supratrochlear artery is located, one of the most common sites for ocular complications. 7 In our previous study, 74 glabellar wrinkle lines were evaluated using Doppler ultrasound. 8 The supratrochlear artery was located lateral to the glabellar wrinkle lines in 44/74 (59%) cases. However, six supratrochlear arteries were located at the glabellar wrinkle line and in the subdermal layer ( Fig. 2 ). In the case of this variation, it is extremely dangerous to inject HA filler at the glabellar wrinkle line.

**Fig. 3:**
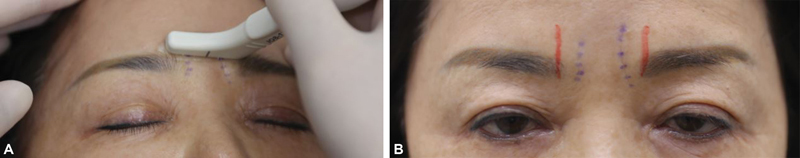
( **A** ) Detection of the supratrochlear artery. ( **B** ) Marking the location of supratrochlear arteries (solid red line) and glabellar wrinkle lines (dotted blue line).

To correct the glabellar wrinkle line, the Doppler ultrasound probe was positioned at the wrinkle line ( Fig. 3A ). The supratrochlear artery is identified and marked in red to show its position relative to the glabellar wrinkle lines ( Fig. 3B ). Considering the rheology in a previous article, Lorient No.2 (HA filler, G' 203 Pa, tan δ 0.2, Joonghun Pharmaceutical, Seoul, Korea) can be injected into the subdermal layer. 9

## Forehead Augmentation

**Fig. 4:**
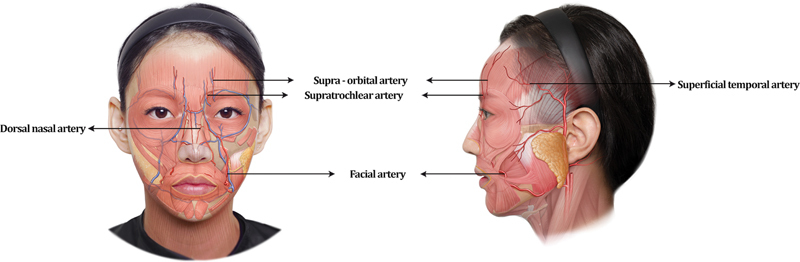
Arteries of the face.

The forehead is a wide area requiring an even injection of HA filler for better outcomes. The forehead area has an extensive network of blood vessels (the supratrochlear artery, supraorbital artery, and frontal branch of the superficial temporal artery; Fig. 4 ). 10

**Fig. 5:**
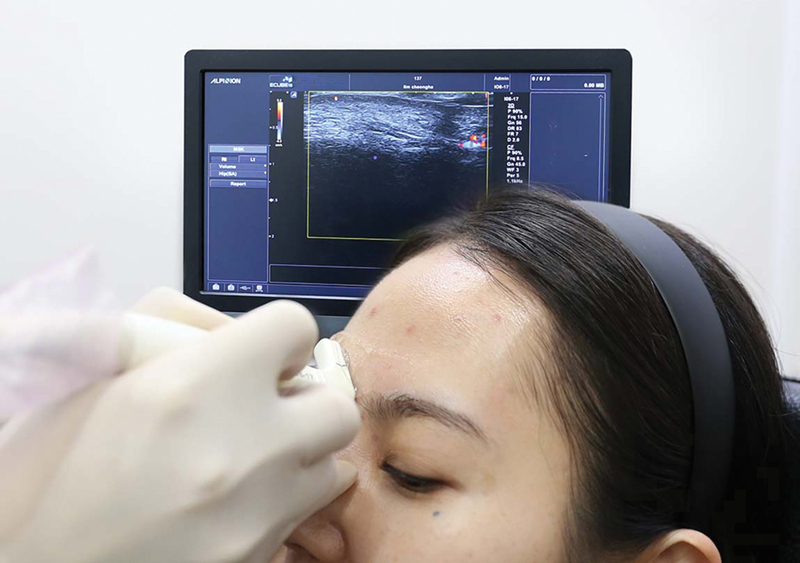
Detection of arteries in the forehead area.

Doppler ultrasound can be used to detect and mark the pathways of these arteries. However, as described in a previous article, the supraorbital artery can exhibit significant variations in its superficial branches and deep branches (18 and 64% of cases, respectively) when detected by Doppler ultrasound. 11 In our study also, the supraorbital artery was relatively difficult to detect. The frontal branch of the superficial temporal artery could be detected more easily in the temple area. After detection of the forehead arterial pathways, HA filler Lorient No.4 (G' 338, tan δ 0.28) was injected based on rheology 9 ( Fig. 5 ).

## Temple Augmentation

**Fig. 6:**
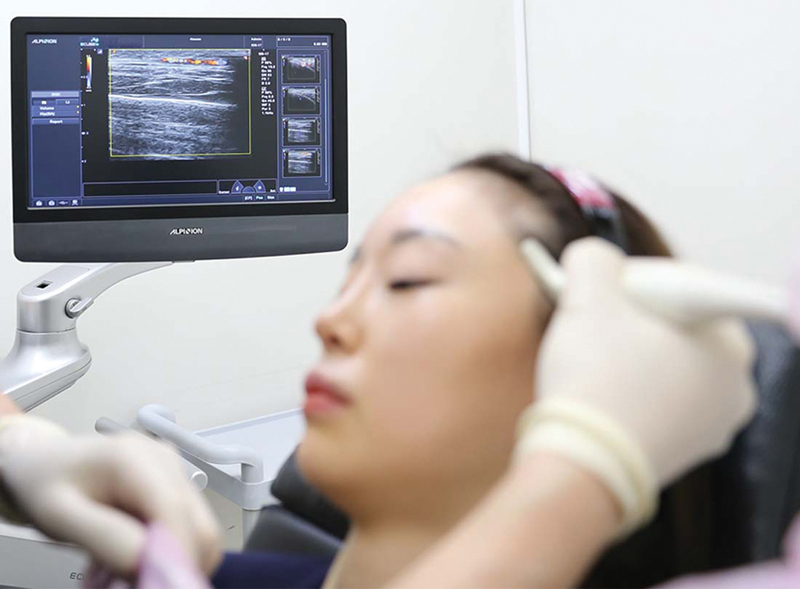
Doppler ultrasound detection of the frontal branch of superficial temporal artery at temple area.

The temple area is composed of complex layers, including the skin, subcutaneous layer, superficial temporal fascia (STF), deep temporal fascia (DTF), and temporalis muscle and bone. 12 The authors recommend injecting HA filler between the STF and DTF. Our previous study showed good results when implementing these guidelines using Lorient No.4 (G' 338, tan δ 0.28, average 1.08 mL on each side). 13 All the procedures were performed after Doppler ultrasound detection ( Fig. 6 ). The frontal branch of the superficial temporal artery is one of the main arteries located in the temple area and can be detected medially, inside, or at the hairline. 14

**Fig. 7:**
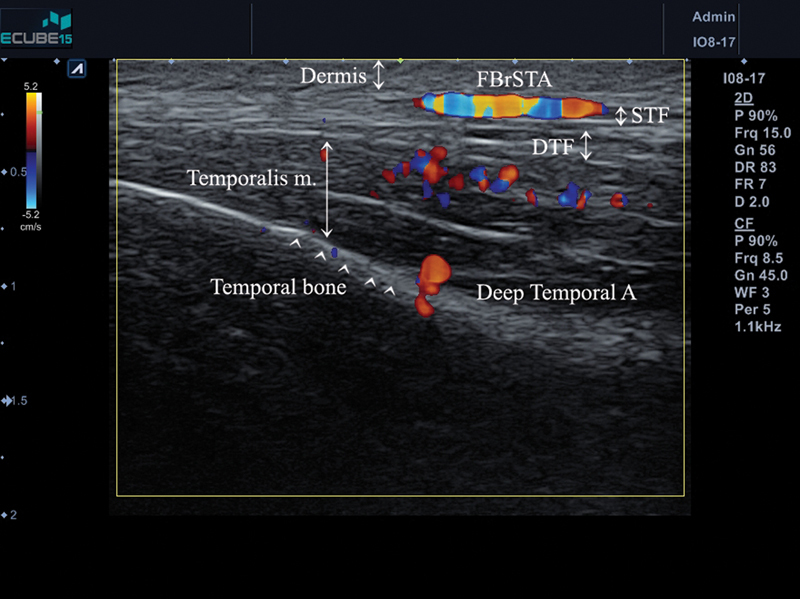
Doppler ultrasound of the temple area. The frontal branch of superficial temporal artery and anterior branch of deep temporal artery are visible.

Another recommendation for temple augmentation involves injecting HA filler into the deep layer, the pre-periosteal layer, by needle. 15 However, our previous study showed that there is a possibility of perforation of the superficial and deep temporal arteries. 16 Our sonographic findings showed that the anterior branch of the deep temporal artery could be interrupted when deeply injected into the temple area ( Fig. 7 ). Therefore, ultrasound scanning prior to injection into the temple area is recommended.

## Nose Augmentation

**Fig. 8:**
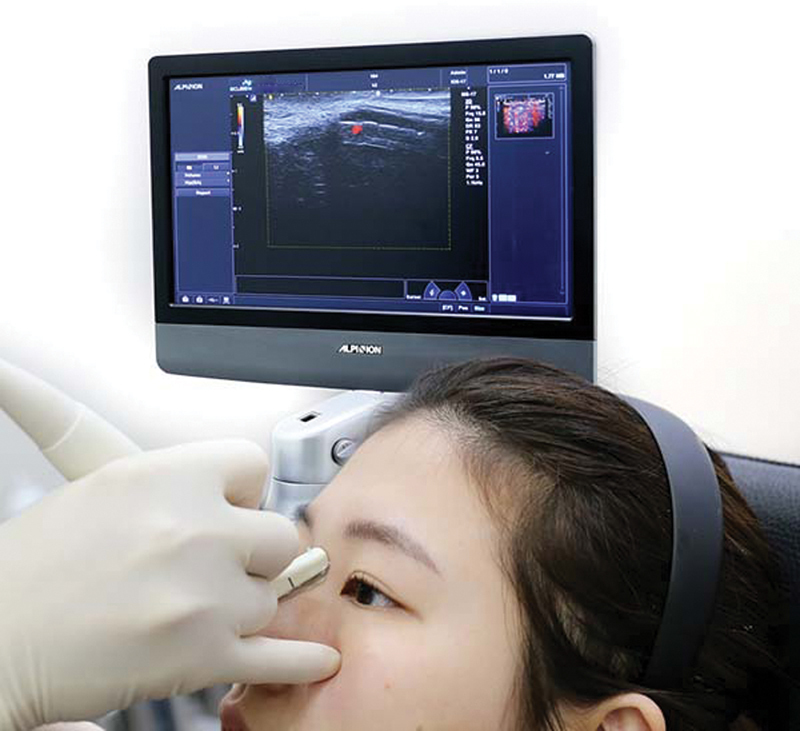
Doppler ultrasound detection at nose.

Nose augmentation by HA filler is one of the most commonly performed procedures, but it also carries its dangers. It is the most common area for ocular complications because the dorsal nasal artery is a branch of the internal carotid artery. 17 In a cadaveric study, arteries and veins were found to run above the fibromuscular layer, so injection at the supraperiosteal layer is determined to be a safe injection plane. 18 However, in our previous study, we showed that some arteries were detected by Doppler ultrasound at the supraperiosteal layer; thus, there was no completely safe layer for injection. 19 The dorsal nasal artery can be detected when the Doppler ultrasound probe is used at the midline of the nose ( Fig. 8 ). The dorsal nasal artery tended to cross the midline of the nose. 20 Therefore, it is recommended to perform Doppler ultrasound before nose injection.

Nose augmentation using HA filler is usually performed using either a cannula or a needle. Our previous study showed that perpendicular injection at the radix area by needle could risk injection of filler into the dorsal nasal artery depending on the needle bevel location. 21 Therefore, cannula injection appears to be a relatively safe procedure. However, the use of a blunt-tip cannula still poses a potential risk. Previous studies have reported that ocular complications were induced by a 25G cannula and even a 23G cannula. 22 According to previous literature, it is relatively safe to perform Doppler ultrasound using a cannula, and gentle injection should be performed for nose augmentation with HA filler. In addition, it is recommended to inject the supraperiosteal layer with a high G' HA filler, such as Lorient No.6 (G' 413, tan δ 0.29).

## Nasolabial Fold Correction

**Fig. 9:**
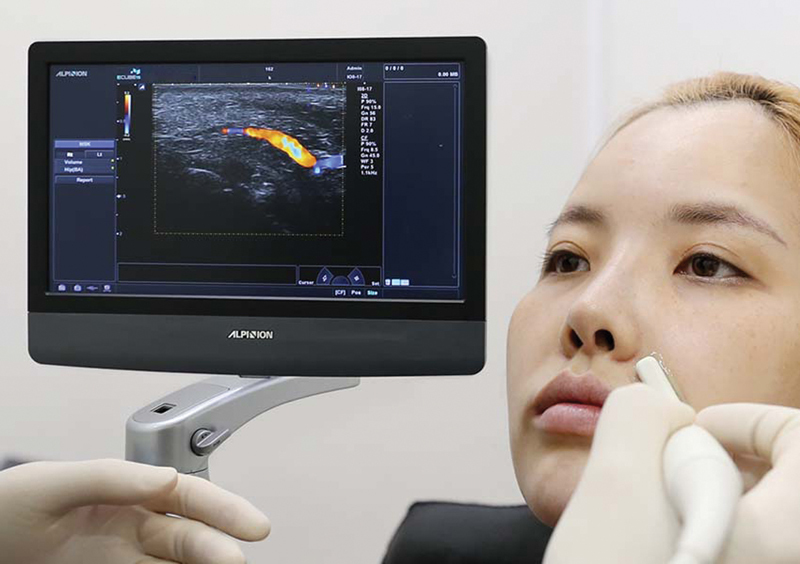
Doppler ultrasound probe being used at the nasolabial fold area. The facial artery is detected at the subcutaneous layer.

Nasolabial fold correction is one of the most common procedures to use filler injections. However, the facial artery runs near the nasolabial fold area and, due to the variation of arterial, can be located under or over muscles. 23 Our previous study showed that the facial artery pathway could be traversing the nasolabial fold area (69%) or lateral to the nasolabial fold area (31%). 24 Therefore, it is relatively safe to detect the facial artery location by Doppler ultrasound first, and then inject high G' HA fillers such as Lorient No.4 or 6 at the supraperiosteal layer ( Fig. 9 ).

## Other Locations for Doppler Ultrasound Detection

Doppler ultrasound can be applied when tear trough correction is performed using an HA filler. The angular artery runs on the medial side of the angular vein. However, the angular artery can vary when approaching the infraorbital artery. 25 The angular vein can be detected by Doppler ultrasound and is useful when injecting HA filler into the tear trough area, and deep injection is recommended. 26 The infraorbital artery can also be detected using Doppler ultrasound and is useful when performing midface augmentation. However, when augmentation of the lateral cheek area, it is relatively difficult to find the transverse facial artery by Doppler ultrasound. HA fillers can also be applied to lower face areas, such as for chin augmentation, lip augmentations, and marionette line correction. The superior and inferior labial arteries can be detected near the lower face area; however, since it is well known that the labial artery tends to run near the mucosal area, filler injection is relatively safe when injected into the vermilion borders of the lips.

## Conclusion




**Video 1**


Doppler ultrasound can be used to detect almost all arteries of the face before injecting the HA filler. The relatively more dangerous sites of filler injection are the glabellar wrinkle, forehead, temple, nose, and nasolabial fold area, and it is recommended to map the vasculature of these areas by Doppler ultrasound before performing filler injection. The Doppler ultrasound detection method is included as a video ( Video 1 , available in the online version). Internal carotid arterial branches, the supratrochlear, supraorbital, and dorsal nasal arteries, and external carotid arterial branches, the superficial temporal and facial arteries, are very important arteries when injecting HA filler; thus, Doppler ultrasound detection is recommended.
